# 72 Multi-Spectral SWIR Imaging in Humans Reveals Correlations with Distinct Skin Burn Depths

**DOI:** 10.1093/jbcr/irad045.046

**Published:** 2023-05-15

**Authors:** Johanna Nunez, Benjamin Levi, Jonathan Hong, Deborah Carlson, Ryan Huebinger, Rodney Chan, Bingchun Wan, Sergey Mironov, Samuel Mandell, Jeremy Goverman, Chiaka Akarichi, Omer Berenfeld, Brett Arnoldo, Audra Clark

**Affiliations:** University of Texas Southwestern, Dallas, Texas; University of Texas Southwestern, Dallas, Texas; University of Texas Southwestern Medical Center, Plano, Texas; UTSW Medical Center, Dallas, Texas; UT Southwestern Med Center, Dallas, Texas; San Antonio Military Medical Center, San Antonio, Texas; UT Southwestern Medical Center, Dallas, Texas; University of Michigan, Ann Arbor, Michigan; Parkland Burn Center / UT Southwestern, Dallas, Texas; Massachusetts General Hospital, Boston, Massachusetts; University of Texas Southwestern/Parkland Burn Center, Dallas, Texas; University of Michigan, Ann Arbor, Michigan; UT Southwestern Medical Center, Dallas, Texas; University of Texas Southwestern, Dallas, Texas; University of Texas Southwestern, Dallas, Texas; University of Texas Southwestern, Dallas, Texas; University of Texas Southwestern Medical Center, Plano, Texas; UTSW Medical Center, Dallas, Texas; UT Southwestern Med Center, Dallas, Texas; San Antonio Military Medical Center, San Antonio, Texas; UT Southwestern Medical Center, Dallas, Texas; University of Michigan, Ann Arbor, Michigan; Parkland Burn Center / UT Southwestern, Dallas, Texas; Massachusetts General Hospital, Boston, Massachusetts; University of Texas Southwestern/Parkland Burn Center, Dallas, Texas; University of Michigan, Ann Arbor, Michigan; UT Southwestern Medical Center, Dallas, Texas; University of Texas Southwestern, Dallas, Texas; University of Texas Southwestern, Dallas, Texas; University of Texas Southwestern, Dallas, Texas; University of Texas Southwestern Medical Center, Plano, Texas; UTSW Medical Center, Dallas, Texas; UT Southwestern Med Center, Dallas, Texas; San Antonio Military Medical Center, San Antonio, Texas; UT Southwestern Medical Center, Dallas, Texas; University of Michigan, Ann Arbor, Michigan; Parkland Burn Center / UT Southwestern, Dallas, Texas; Massachusetts General Hospital, Boston, Massachusetts; University of Texas Southwestern/Parkland Burn Center, Dallas, Texas; University of Michigan, Ann Arbor, Michigan; UT Southwestern Medical Center, Dallas, Texas; University of Texas Southwestern, Dallas, Texas; University of Texas Southwestern, Dallas, Texas; University of Texas Southwestern, Dallas, Texas; University of Texas Southwestern Medical Center, Plano, Texas; UTSW Medical Center, Dallas, Texas; UT Southwestern Med Center, Dallas, Texas; San Antonio Military Medical Center, San Antonio, Texas; UT Southwestern Medical Center, Dallas, Texas; University of Michigan, Ann Arbor, Michigan; Parkland Burn Center / UT Southwestern, Dallas, Texas; Massachusetts General Hospital, Boston, Massachusetts; University of Texas Southwestern/Parkland Burn Center, Dallas, Texas; University of Michigan, Ann Arbor, Michigan; UT Southwestern Medical Center, Dallas, Texas; University of Texas Southwestern, Dallas, Texas; University of Texas Southwestern, Dallas, Texas; University of Texas Southwestern, Dallas, Texas; University of Texas Southwestern Medical Center, Plano, Texas; UTSW Medical Center, Dallas, Texas; UT Southwestern Med Center, Dallas, Texas; San Antonio Military Medical Center, San Antonio, Texas; UT Southwestern Medical Center, Dallas, Texas; University of Michigan, Ann Arbor, Michigan; Parkland Burn Center / UT Southwestern, Dallas, Texas; Massachusetts General Hospital, Boston, Massachusetts; University of Texas Southwestern/Parkland Burn Center, Dallas, Texas; University of Michigan, Ann Arbor, Michigan; UT Southwestern Medical Center, Dallas, Texas; University of Texas Southwestern, Dallas, Texas; University of Texas Southwestern, Dallas, Texas; University of Texas Southwestern, Dallas, Texas; University of Texas Southwestern Medical Center, Plano, Texas; UTSW Medical Center, Dallas, Texas; UT Southwestern Med Center, Dallas, Texas; San Antonio Military Medical Center, San Antonio, Texas; UT Southwestern Medical Center, Dallas, Texas; University of Michigan, Ann Arbor, Michigan; Parkland Burn Center / UT Southwestern, Dallas, Texas; Massachusetts General Hospital, Boston, Massachusetts; University of Texas Southwestern/Parkland Burn Center, Dallas, Texas; University of Michigan, Ann Arbor, Michigan; UT Southwestern Medical Center, Dallas, Texas; University of Texas Southwestern, Dallas, Texas; University of Texas Southwestern, Dallas, Texas; University of Texas Southwestern, Dallas, Texas; University of Texas Southwestern Medical Center, Plano, Texas; UTSW Medical Center, Dallas, Texas; UT Southwestern Med Center, Dallas, Texas; San Antonio Military Medical Center, San Antonio, Texas; UT Southwestern Medical Center, Dallas, Texas; University of Michigan, Ann Arbor, Michigan; Parkland Burn Center / UT Southwestern, Dallas, Texas; Massachusetts General Hospital, Boston, Massachusetts; University of Texas Southwestern/Parkland Burn Center, Dallas, Texas; University of Michigan, Ann Arbor, Michigan; UT Southwestern Medical Center, Dallas, Texas; University of Texas Southwestern, Dallas, Texas; University of Texas Southwestern, Dallas, Texas; University of Texas Southwestern, Dallas, Texas; University of Texas Southwestern Medical Center, Plano, Texas; UTSW Medical Center, Dallas, Texas; UT Southwestern Med Center, Dallas, Texas; San Antonio Military Medical Center, San Antonio, Texas; UT Southwestern Medical Center, Dallas, Texas; University of Michigan, Ann Arbor, Michigan; Parkland Burn Center / UT Southwestern, Dallas, Texas; Massachusetts General Hospital, Boston, Massachusetts; University of Texas Southwestern/Parkland Burn Center, Dallas, Texas; University of Michigan, Ann Arbor, Michigan; UT Southwestern Medical Center, Dallas, Texas; University of Texas Southwestern, Dallas, Texas; University of Texas Southwestern, Dallas, Texas; University of Texas Southwestern, Dallas, Texas; University of Texas Southwestern Medical Center, Plano, Texas; UTSW Medical Center, Dallas, Texas; UT Southwestern Med Center, Dallas, Texas; San Antonio Military Medical Center, San Antonio, Texas; UT Southwestern Medical Center, Dallas, Texas; University of Michigan, Ann Arbor, Michigan; Parkland Burn Center / UT Southwestern, Dallas, Texas; Massachusetts General Hospital, Boston, Massachusetts; University of Texas Southwestern/Parkland Burn Center, Dallas, Texas; University of Michigan, Ann Arbor, Michigan; UT Southwestern Medical Center, Dallas, Texas; University of Texas Southwestern, Dallas, Texas; University of Texas Southwestern, Dallas, Texas; University of Texas Southwestern, Dallas, Texas; University of Texas Southwestern Medical Center, Plano, Texas; UTSW Medical Center, Dallas, Texas; UT Southwestern Med Center, Dallas, Texas; San Antonio Military Medical Center, San Antonio, Texas; UT Southwestern Medical Center, Dallas, Texas; University of Michigan, Ann Arbor, Michigan; Parkland Burn Center / UT Southwestern, Dallas, Texas; Massachusetts General Hospital, Boston, Massachusetts; University of Texas Southwestern/Parkland Burn Center, Dallas, Texas; University of Michigan, Ann Arbor, Michigan; UT Southwestern Medical Center, Dallas, Texas; University of Texas Southwestern, Dallas, Texas; University of Texas Southwestern, Dallas, Texas; University of Texas Southwestern, Dallas, Texas; University of Texas Southwestern Medical Center, Plano, Texas; UTSW Medical Center, Dallas, Texas; UT Southwestern Med Center, Dallas, Texas; San Antonio Military Medical Center, San Antonio, Texas; UT Southwestern Medical Center, Dallas, Texas; University of Michigan, Ann Arbor, Michigan; Parkland Burn Center / UT Southwestern, Dallas, Texas; Massachusetts General Hospital, Boston, Massachusetts; University of Texas Southwestern/Parkland Burn Center, Dallas, Texas; University of Michigan, Ann Arbor, Michigan; UT Southwestern Medical Center, Dallas, Texas; University of Texas Southwestern, Dallas, Texas; University of Texas Southwestern, Dallas, Texas; University of Texas Southwestern, Dallas, Texas; University of Texas Southwestern Medical Center, Plano, Texas; UTSW Medical Center, Dallas, Texas; UT Southwestern Med Center, Dallas, Texas; San Antonio Military Medical Center, San Antonio, Texas; UT Southwestern Medical Center, Dallas, Texas; University of Michigan, Ann Arbor, Michigan; Parkland Burn Center / UT Southwestern, Dallas, Texas; Massachusetts General Hospital, Boston, Massachusetts; University of Texas Southwestern/Parkland Burn Center, Dallas, Texas; University of Michigan, Ann Arbor, Michigan; UT Southwestern Medical Center, Dallas, Texas; University of Texas Southwestern, Dallas, Texas; University of Texas Southwestern, Dallas, Texas; University of Texas Southwestern, Dallas, Texas; University of Texas Southwestern Medical Center, Plano, Texas; UTSW Medical Center, Dallas, Texas; UT Southwestern Med Center, Dallas, Texas; San Antonio Military Medical Center, San Antonio, Texas; UT Southwestern Medical Center, Dallas, Texas; University of Michigan, Ann Arbor, Michigan; Parkland Burn Center / UT Southwestern, Dallas, Texas; Massachusetts General Hospital, Boston, Massachusetts; University of Texas Southwestern/Parkland Burn Center, Dallas, Texas; University of Michigan, Ann Arbor, Michigan; UT Southwestern Medical Center, Dallas, Texas; University of Texas Southwestern, Dallas, Texas; University of Texas Southwestern, Dallas, Texas; University of Texas Southwestern, Dallas, Texas; University of Texas Southwestern Medical Center, Plano, Texas; UTSW Medical Center, Dallas, Texas; UT Southwestern Med Center, Dallas, Texas; San Antonio Military Medical Center, San Antonio, Texas; UT Southwestern Medical Center, Dallas, Texas; University of Michigan, Ann Arbor, Michigan; Parkland Burn Center / UT Southwestern, Dallas, Texas; Massachusetts General Hospital, Boston, Massachusetts; University of Texas Southwestern/Parkland Burn Center, Dallas, Texas; University of Michigan, Ann Arbor, Michigan; UT Southwestern Medical Center, Dallas, Texas; University of Texas Southwestern, Dallas, Texas

## Abstract

**Introduction:**

Burn depth determination is a critical aspect of burn patient care but currently lacks accuracy in clinical practice. We have shown first in animal models and then in a two-patient characterization that Short Wave Infrared (SWIR) imaging, which penetrates tissue better than visual or near-infrared light and is very sensitive to water content, can distinguish between superficial and deep tissue necrosis. Here we present the findings from our pilot study of 10 patients showing the use of multi-spectral SWIR imaging of human burn injury as a potential technique for burn depth determination.

**Methods:**

Ten patients admitted for mixed depth thermal injuries between 5-40% TBSA were analyzed using our SWIR assessment tool. Prior to burn excision the SWIR system was used to image burn areas and normal skin at 4 different SWIR wavelengths (**Figure 1A**). Standard photographs from imaged areas were collected and presented for 5 independent, blinded, surgeons’ assessments. Using the visual and SWIR images, 5-15 regions of interest (ROI) were selected from each of the burned areas and normalized to adjacent normal skin and the reflected light intensity in each ROI was averaged at each wavelength. Statistics were done using Mann-Whitney U tests.

**Results:**

Visual and SWIR images were collected from burn areas in 10 patients for a total of 273 burn ROIs. The ROIs were assessed by the surgeons with ROIs being agreed upon as being superficial or superficial partial thickness (SPT) (n=47), deep partial thickness (DPT) (n=97), or full thickness (FT) (n=129) burns by a majority (60% or above) consensus. As seen in **Figure 1B**, the reflectance intensities (RIs) from superficial and SPT burns were significantly different than the DPT burns at the 1940 nm wavelength (p=0.05) and significantly different than FT burns at 1200 nm (p=0.008) and 1940 nm (p=0.009). When DPT and FT burns were compared there was a significant difference a 1200 nm (p=0.003) and 2250 nm (p=0.01).

**Conclusions:**

Here we present a 10 patient SWIR pilot study demonstrating distinct ROIs of SWIR wavelengths for different burn depths based on consensus surgeon assessments. Overall, as burn depth increases, the 1200 and 2250 nm wavelengths show increasing RIs and 1940 nm show decreasing RIs. These results motivate further studies of SWIR imaging including pending histological analysis in the hope to non-invasively and accurately identify operative versus non-operative burns.

**Applicability of Research to Practice:**

This new SWIR technology may enable us to more objectively assess burn depth, leading to improved surgical decision making and better patient outcomes.

